# Single center two years’ experience of Ozaki procedure: Early follow-up

**DOI:** 10.1097/MD.0000000000035935

**Published:** 2023-11-10

**Authors:** Yasin Ozden, Safa Ozcelik, Kemal Ozdemir, Ferdi Peynirci, Seyma Ozden, Mutlu Senocak, Yavuz Sensoz, Ilyas Kayacioglu,

**Affiliations:** a Department of Cardiovascular Surgery, Dr Siyami Ersek Thoracic and Cardiovascular Surgery Training and Research Hospital, Istanbul, Turkey; b Department of Cardiovascular Surgery, Kartal Kosuyolu High Speciality Training and Research Hospital, Istanbul, Turkey; c Department of Chest Diseases, Immunology and Allergy Clinic, Sureyyapasa Chest Diseases and Thoracic Surgery Training and Research Hospital, Istanbul, Turkey.

**Keywords:** aortic valve insufficiency, aortic valve neocuspidization, aortic valve stenosis, aortic valve surgery, ozaki procedure

## Abstract

The Ozaki Procedure is an innovative surgical technique aiming of aortic valve neocuspidization using glutaraldehyde-treated autologous pericardium was first developed by Ozaki et al in 2007. With this newly developed technique, valve replacement was achieved without using prosthetic material due to both aortic stenosis and aortic insufficiency. Between December 2020 and December 2022, a total of 59 patients were operated on with the Ozaki Procedure due to aortic valve pathologies in our center. We evaluated the pre- and postoperative as well as the first-month data of a total of 44 patients with isolated the Ozaki Procedure and compared their echocardiographic changes. Patients with isolated aortic valve pathology were included in the study. Fifteen patients who underwent simultaneous coronary artery bypass surgery and Ozaki Procedure were excluded from the analysis. In the first month after the operation, n:2 (%4.5) patients died. When the preoperative and postoperative 1st month echocardiographic data of the remaining patients were compared, it was found that the decrease in mean gradient, max gradient and peak velocity values in the aortic valve was statistically significant. This is due to the fact that reaching neo-valves has very similar hemodynamics to the native aortic valve. Aortic valve neocuspidization by Ozaki Procedure may be a viable alternative to both surgical aortic valve replacement (AVR) and transcatheter aortic valve implantation. Its popularity and application is increasing all over the world. Short and mid-term results are available in the literature. The short and mid-term results are good, and the long-term results are hopeful.

## 1. Introduction

Aortic valve pathologies are a serious public health problem with an increasing frequency in terms of valve diseases.^[1]^ And conventional open valve replacement is still the gold standard in its treatment.^[2]^ For this situation, mechanical and biological prosthetic valves are used. Besides, a new technique described by Ozaki has emerged in the last 15 years. This technique is aortic valve neocuspidization (AV-Neo) using glutaraldehyde-treated autologous pericardium.^[3]^

The Ozaki Procedure, that is, an innovative surgical technique aiming of AV-Neo using glutaraldehyde-treated autologous pericardium was first developed by Ozaki et al in 2007.^[3]^ Although not very successful results have been achieved, the use of non-treated autologous pericardium for aortic valve reconstruction dates back to the 1960s.^[4,5]^

Mitral and tricuspid valve repair methods are very popular in the world recently. Besides, the gold standard treatment for aortic valve is still aortic valve replacement (AVR). However, despite advances in the design and construction of prosthetic valves, hemodynamic performance and valve gradient values are not yet as adequate as natural aortic valves. The most important advantage of the Ozaki technique is that it obtains hemodynamic parameters closest to the native aortic valve. Since the technique is sutured to the natural annulus, postoperative central aortic insufficiency may occur, but there is no residual aortic stenosis. Another favorable aspect is that no foreign material other than sutures is used. Therefore, there is no need for lifelong anticoagulant medication. The biggest disadvantage is that it is not known how long the neo-valve will degenerate.^[6]^

Thanks to this new aortic valve neocuspidization technique developed by Ozaki, it can be applied in cases of both aortic stenosis, aortic regurgitation and aortic valve infective endocarditis in both tricuspid and bicuspid aortic valves. There are no absolute contraindications for this method. In cases where the pericardium is too thin or the pericardium is not durable enough, such as pericarditis, this method can be applied using animal pericardium.^[7]^

The main aim of our study was to demonstrate the efficacy of the Ozaki Procedure and to show that the high-pressure gradients in the aortic valve decreased rapidly even at 1 month postoperatively.

## 2. Materials and Methods

**Figure 1.**
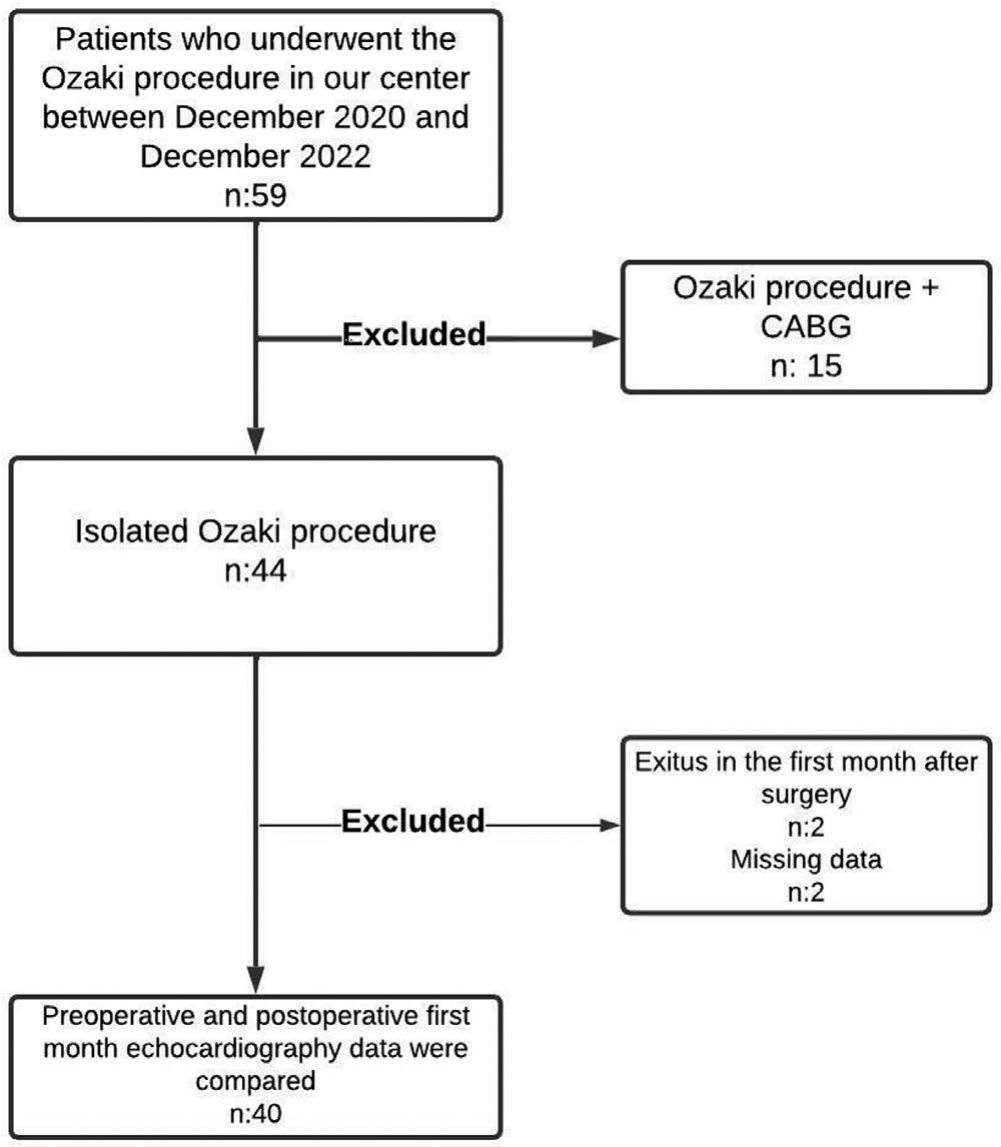
Flow chart of study.

We performed a retrospective, single-center study. The Ozaki Procedure technique was first applied in our hospital in December 2020. As of this date, between December 2020 and December 2022, a total of 59 patients were operated with the Ozaki Procedure. Of these patients, 44 were operated on with the isolated Ozaki Procedure, and 15 were those in whom coronary artery bypass grafting (CABG) surgery was performed in combination with the Ozaki Procedure. In this study, we only considered patients who underwent the isolated Ozaki Procedure. Patients treated in combination with CABG were excluded from the study (Fig. 1). Because CABG is another cardiac operation in itself and may lead to unique outcomes and significant changes in operative and postoperative parameters of patients.

No distinction was made between stenosis or insufficiency in patient selection. All patients in whom we performed the Ozaki Procedure were equivalent to patients with a previous indication for AVR. If a patient had an indication for AVR for any pathology, we performed the Ozaki Procedure in these patients. None of the patients had infective endocarditis. Autologous pericardium was used in all of the patients we operated on. No homologous or animal pericardium was used. None of our patients had ever had pericarditis. All patients underwent thoracic computed tomography for pericardial thickening and adhesions.

Preoperative clinical data, demographic data, operative data and postoperative clinical data of the remaining 44 patients were collected by retrospective file analysis. All patients in whom we performed isolated Ozaki Procedure were included in the study. Patients with low ejection fraction or non-cardiac pathologies (diabetes mellitus, elevated creatinine levels, carotid artery lesions and previous coronary artery intervention) were not excluded. Preoperative and postoperative echocardiograms in first month were compared.

### 2.1. Surgical Technique

**Figure 2.**
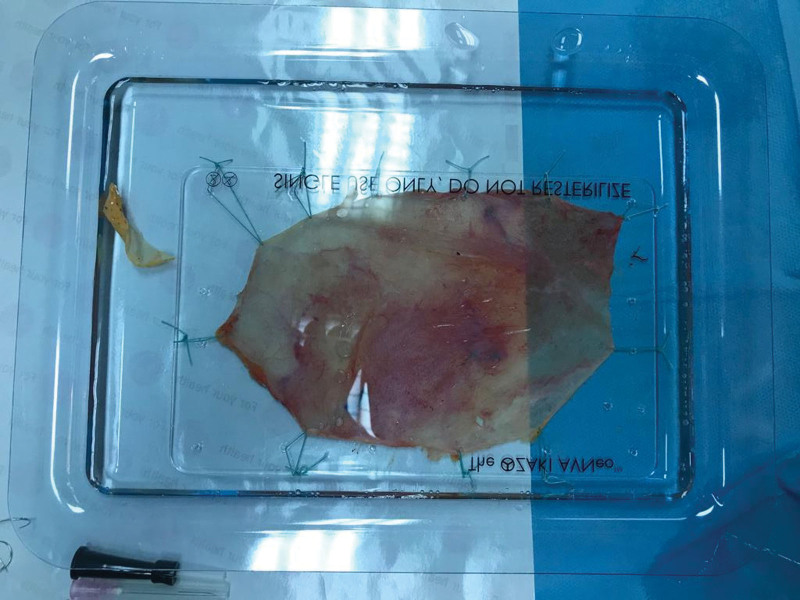
Autologous pericardial graft fixed on the plate by stretching its edges.

A standard median sternotomy incision is performed. After sternotomy, the fat and fibrous tissues on the parietal surface of the pericardium are cleaned and an area of approximately 7 × 8 cm is prepared on the pericardium. The diaphragmatic side is marked and the pericardial graft is removed with only scissors without using cautery. The removed pericardial graft is stretched from its edges and fixed on the plate (Fig. 2). The pericardium, which is kept in 0.6% glutaraldehyde solution for 10 minutes, is removed from the fixed plate. Then, it is washed by keeping it in isotonic solutions for 3 times - 6 minutes.

**Figure 3.**
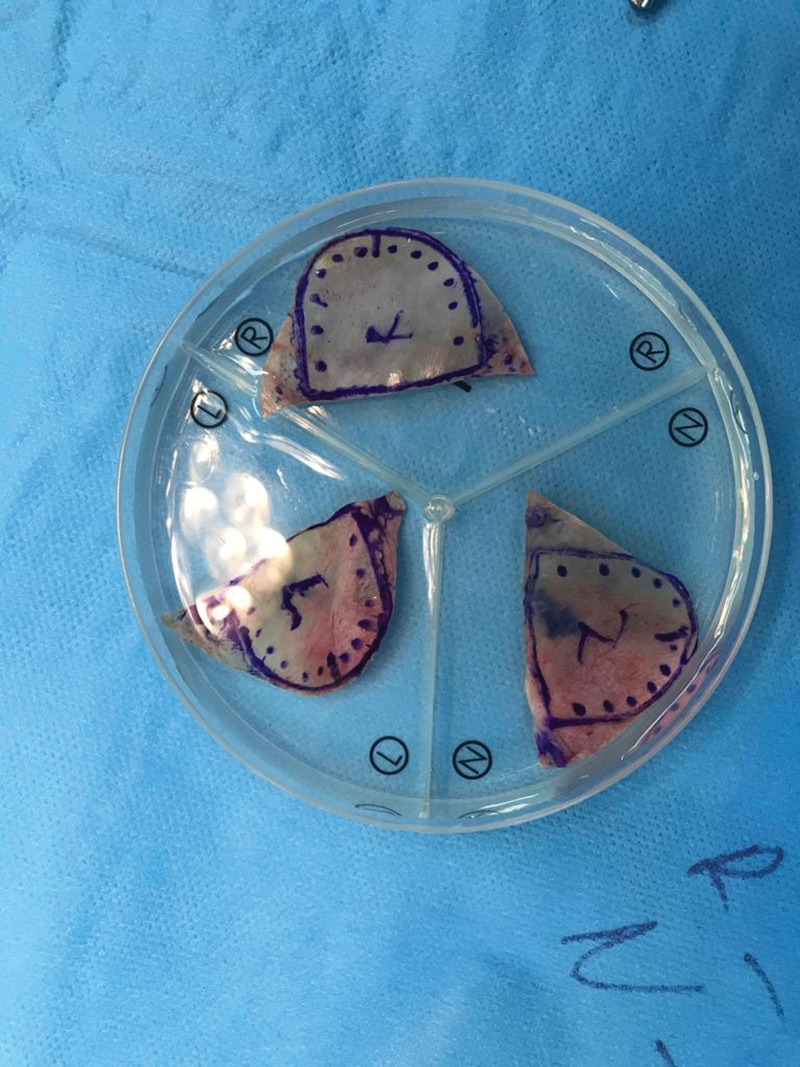
New aortic valve leaflets prepared according to aortic annulus measurements.

The surgical procedure is performed under cardiopulmonary bypass and aortic cross-clamp. Aortotomy is performed transversely approximately 1.5–2 cm distal to the right coronary orifice. The native aortic valve and all calcifications are excised without compromising the integrity of the annulus. Each inter-commissural distances are measured with the help of original apparatus developed by Ozaki. During the measurement, the midpoint of each inter-commissural distance is also marked with an indelible pen. An independent measure is calculated for each leaflet. If you are stuck between 2 sizes, the bigger size is used. According to the calculated measurements, the leaflets and the points to be sutured are drawn with the sets developed by Ozaki from the visceral side of the pericardium. As much as possible, a marked surface close to the diaphragm is used. Because this is the thickest surface of the pericardium. The pericardium is cut with scissors on both sides with the wing suture point inside, and each leaflet is prepared separately (Fig. 3).

The neocusps are sutured to the annular ring in right, left and non-coronary order. But this is not mandatory. In some cases, we started from the left coronary cusp. 4-0 polypropylene sutures are used. Neocups are placed with the visceral side facing the ventricle. First, the midpoint of the intercommissural and neocusp midpoint are crossed and the neocusp is tied under the annulus. Suturation; it is made from the aortic side to the ventricular surface on the neocusp, and from the ventricle side to the aortic side on the annulus. In the first 3 sutures, the annulus/neocusp suture transition ratio becomes 1/3, then 1/1 after the remaining distance is equalized. Continuous suturing ends at the commissure and the suture is exited 2 to 3 mm below the commissure of the aorta. The penultimate suture is the suture called the “big bite” and is passed deeper and thicker than the annulus.

**Figure 4.**
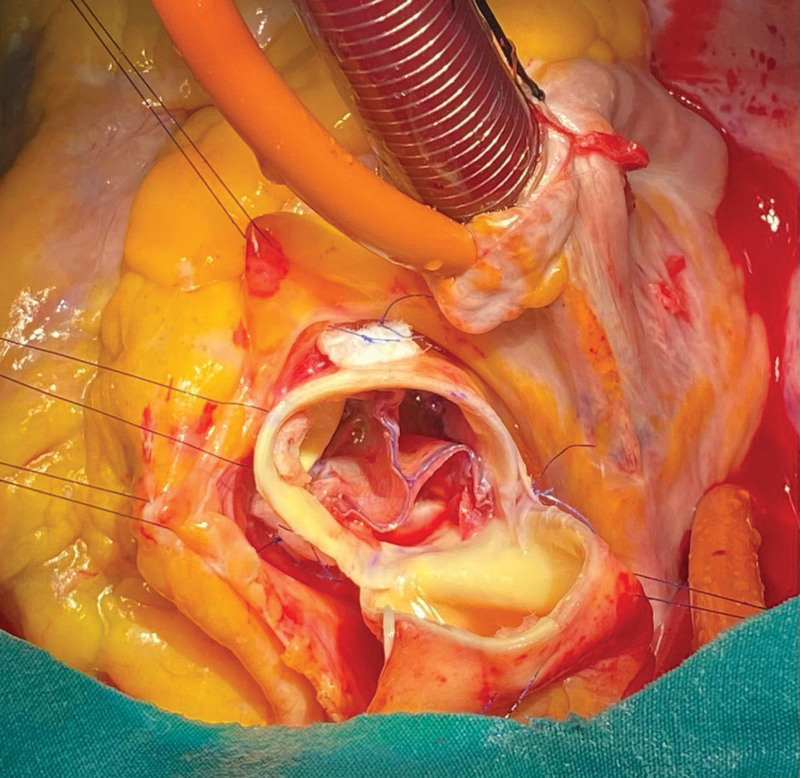
Ozaki Procedure on preoperative tricuspid aortic valve: Postoperative Windmill View.

**Figure 5.**
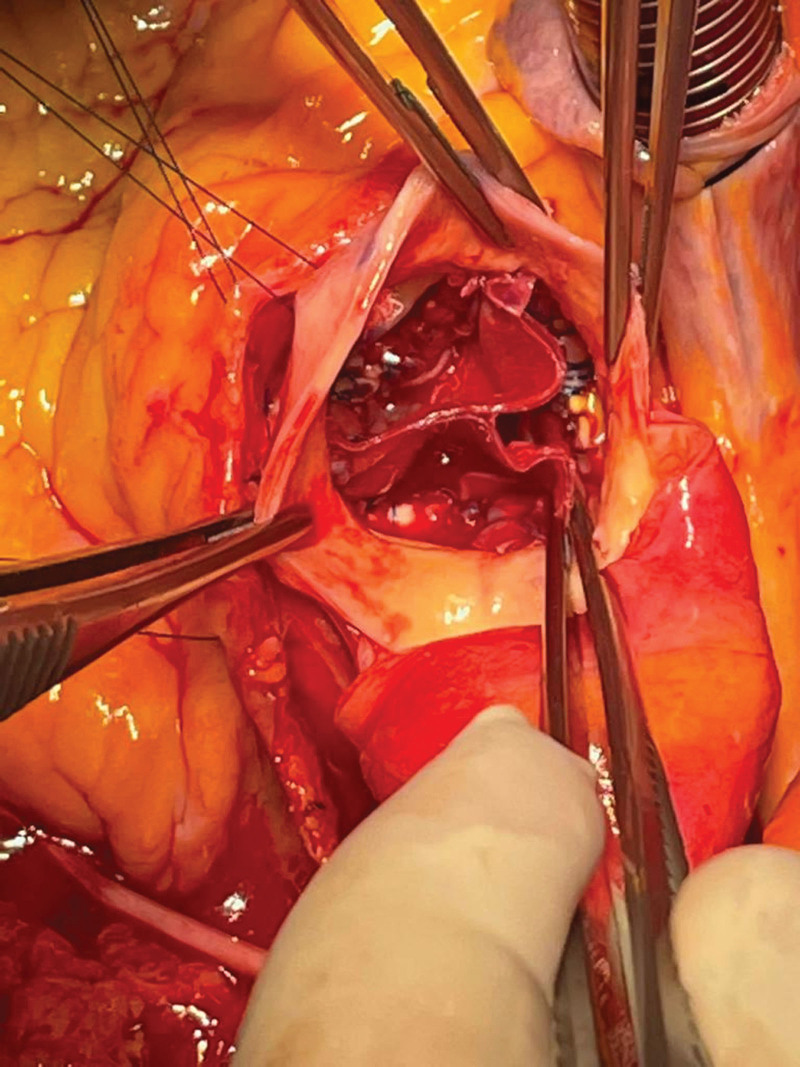
Ozaki Procedure on preoperative bicuspid aortic valve: Postoperative Windmill View.

**Figure 6.**
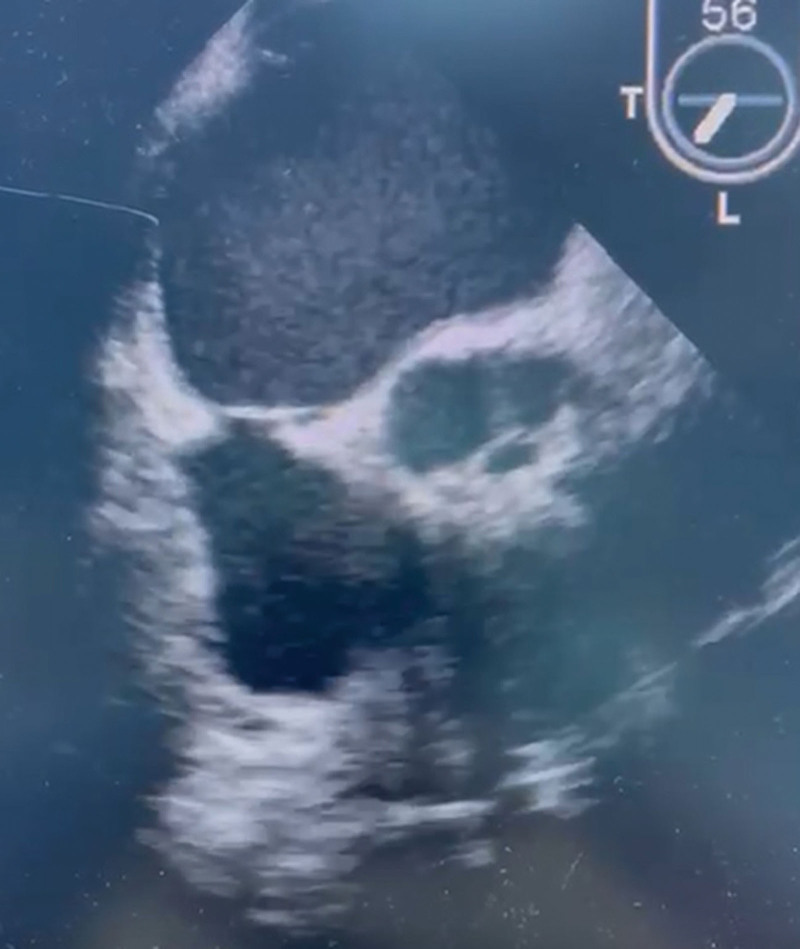
Postoperative transesophageal echocardiography image.

All neocusps are completed in the same way. A new 4-0 suture is passed through the corners of the adjacent neocusps and then the wings, and the suture is taken out of the aorta 2–3 mm above the commissure so that the wings of each neocusp adhere to the aortic wall. For each commissure, there should be a total of 4 sutures, 2 at the top and 2 at the bottom. These sutures are fixed outside the commissure with a 5 × 10 mm wide plejit. And thus, a new valve is formed in the form of a weather vane (Figs. 4 and 5). After weaning from cardiopulmonary bypass, neovalve coaptation is checked with a transesophageal echocardiogram (Fig. 6).

### 2.2. Statistical analysis

Statistical analyzes of the study were performed using the SPSS 22.0 (SPSS Inc., Chicago, IL) package program which is trial version. Quantitative variables are suitable for normal distribution examined by Kolmogorov-Smirnov. Dependent measures conforming to the normal distribution with the paired t test, dependent measures that do not conform to the normal distribution are Wilcoxon Examined by *t* test. Quantitative conforming to a normal distribution descriptive statistics of variables mean ± standard deviation, non-normally distributed quantitative The descriptive statistics of the variables were shown as the median (25–75th percentile). Qualitative Descriptive statistics for the variables were expressed as frequency (%). *P* < .05 values were considered statistically significant.

### 2.3. Declarations

Study approval was obtained from the institution scientific committee. This study was conducted in accordance with the Declaration of Helsinki. Individual consent for the study was waived because of the retrospective nature of the study and because the analysis used anonymous clinical data.

## 3. Results

During the study, a total of 44 patients were operated on with the Ozaki Procedure (16 male—28 female). The mean age was 65.66 years 21 of the patients had diabetes mellitus and the mean HbA1c of all patients was 6.21. None of the patients were on dialysis and the mean creatinine was 0.86 mg/dL. Three patients had previously undergone coronary artery intervention and none of them had undergone cardiac surgery. None of the patients had severe carotid artery stenosis (Table 1). Preoperative baseline characteristics of patients are depicted in Table 1.

**Table 1:** Preoperative baseline characteristics.

Variables	All patients (n = 44)
Gender, Female, n (%)	28 (63.6)
Age (yr), mean ± SD	65.66 ± 11.316
Creatinine (mg/dL), mean ± SD	0.86 ± 0.203
Glomerular filtration rate (mL/min/1.73 m^2^), mean ± SD	79.55 ± 17.345
Patient on dialysis, n (%)	0 (0)
Diabetes mellitus, n (%)	21 (47.7)
HbA1c, mean ± SD	6.21 ± 0.784
Patient undergoing preoperative angiographic coronary interventional procedure, n (%)	3 (6.8)
Coronary artery stenosis with doppler diagnosis, n (%)	
Normal	6 (13.6)
Minimal atherosclerotic plaque	35 (79.5)
Unilateral < 70% stenosis	2 (4.5)
Bilateral < 70% stenosis	1 (2.3)
Bilateral > 70% stenosis	0 (0)
Previous cardiac operation, n (%)	0 (0)

Surgery was planned for 39 of these patients due to severe aortic stenosis and 5 due to severe aortic regurgitation pathology. Twelve of the patients with severe aortic stenosis had combined moderate or severe aortic regurgitation. Two of the patients with severe aortic regurgitation had combined moderate or severe aortic stenosis. Two patients had bicuspid valve. None of the patients had a unicuspid valve.

According to Euroscore II, 10 of the patients were at moderate risk for the operation in preoperative period. There were no high-risk patients (Table 2).

**Table 2:** Operative data.

Variables	All patients (n = 44)
Euroscore II score, Mean ± SD	1.56 ± 1.092
Risk status according to Euroscore II, n (%)	
Low risk	34 (77.3)
Moderate risk	10 (22.7)
High risk	0 (0)
Cardiopulmonary bypass time (min), Mean ± SD	148.00 ± 33.465
Cross-clamp time (min), Mean ± SD	114.00 ± 21.316

Our mean cardiopulmonary bypass and cross-clamp times for the operations were 148.00 ± 33.465 and 114.00 ± 21.316 minutes, respectively.

In the postoperative 1 month period, 2 of our patients died due to procedure. One died on the 4th postoperative day and the other on the 13th postoperative day. Both patients died due to multiorgan failure due to prolonged cross-clamp times.

None of the patients needed an intra-aortic balloon pump or extracorporeal membrane oxygenation. Hemofiltration was performed in only 2 patients, and they were exitus patients. In the postoperative period, 6 patients underwent revision surgery in terms of pericardial effusion, tamponade or cardiac low flow. Two of them were patients who died. Only 1 patient was operated in another hospital for severe aortic regurgitation.

No patient was routinely started on warfarin. Only 100 mg of acetylsalicylic acid was routinely used for all patients. Warfarin was started only in patients who developed postoperative atrial fibrillation (19 patients). As a result, 1 patient had cerebrovascular disease on the 3rd postoperative day.

Forty patients (90.9%) could be extubated within the first 24 hours and the average hospital stay was 10 days (Table 3).

**Table 3:** Postoperative data.

Variables	All patients (n = 44)
IABP, n (%)	0 (0)
ECMO, n (%)	0 (0)
Hemofiltration, n (%)	2 (4.5)
Revision reoperation	6 (13.6)
SVD, n (%)	1 (2.3)
Ex, n (%)	2 (4.5)
Extubation time, n (%)	
<12 h	40 (90.9)
12–24 h	1 (2.3)
>24 h	1 (2.3)
Non extübe/ex/tx	2 (4.5)
ICU time, n (%)	
0–24 h	13 (29.5)
>24 h	31 (70.5)
Day of hospitalization, Mean ± SD	10.09 ± 6.420
Postop AF, Mean ± SD	19 (43.2)

AF = atrial fibrillation, ECMO = extracorporeal membrane oxygenation, ICU = intensive care unit, IABP = intra aortic balloon pump.

Preoperative and postoperative 1st month echocardiographic data of the patients were examined. According to preoperative data, the mean EF was 55.80%. Nine patients had moderate mitral regurgitation and 1 patient had severe mitral regurgitation. None of them had organic mitral valve pathology and no surgical intervention was performed on the mitral valve (Table 4). In the postoperative 1st month, mitral regurgitation persisted in the patient with severe mitral regurgitation and decreased in 6 patients out of 9 patients with moderate mitral regurgitation. This was because when aortic valve pathology was corrected in patients without organic mitral valve pathology, mitral valve regurgitation was automatically corrected. Only 1 patient with severe aortic regurgitation and 2 patients with moderate aortic regurgitation remained persistent postoperatively. No residual aortic stenosis was observed in any patient (Table 5).

**Table 4:** Preoperative patients echocardiographic data.

Variables	All patients (n = 44)
EF (%), Mean ± SD	55.80 ± 8.953
MR, n (%)	
None	10 (22.7)
Mild	24 (54.5)
Moderate	9 (20.5)
Severe	1 (2.3)
MS, n (%)	
None	41 (93.2)
Mild	3 (6.8)
Moderate	0 (0)
Severe	0 (0)
AR, n (%)	
None	12 (27.3)
Mild	14 (31.8)
Moderate	12 (27.3)
Moderate - Severe	1 (2.3)
Severe	5 (11.4)
AS, n (%)	
None	3 (6.8)
Mild	1 (2.3)
Moderate	1 (2.3)
Severe	39 (88.6)
Aortic Mean Gradient (mm Hg), Mean ± SD	46.88 ± 15.845
Aortic Max Gradient (mm Hg), Mean ± SD	75.79 ± 24.565
Aortic vMax (m/sec), Mean ± SD	4.25 ± 0.807
PAP (mm Hg), Mean ± SD	33.25 ± 11.200
PHT, n (%)	
None or Mild (0–30 mm Hg)	21 (47.7)
Moderate (31–55 mm Hg)	19 (43.2)
Severe (> 55 mm Hg)	4 (9.1)
Low EF low gradient, n (%)	
No	43 (97.7)
Yes	1 (2.3)

AR = aortic regurgitation, AS = aortic stenosis, EF = ejection fraction, MR = mitral regurgitation, MS = mitral stenosis, PAP = pulmonary artery pressure, PHT = pulmonary hypertension.

**Table 5:** Postoperative patients echocardiographic data.

Variables	All patients (n = 40)
EF (%), Mean ± SD	54.25 ± 7.641
MR, n (%)	
None	9 (20.5)
Mild	27 (61.4)
Moderate	3 (6.8)
Severe	1 (2.3)
AR, n (%)	
None	24 (54.5)
Mild	13 (29.5)
Moderate	2 (4.5)
Severe	1 (2.3)
AS, n (%)	
None	40 (100)
Aortic Mean Gradient (mm Hg), Mean ± SD	8.33 ± 3.189
Aortic Max Gradient (mm Hg), Mean ± SD	16.10 ± 5.757
Aortic vMax (m/sec), Mean ± SD	1.90 ± 0.353
PAP (mm Hg), Mean ± SD	30.88 ± 11.822
PHT, n (%)	
None or Mild (0–30 mm Hg)	24 (54.5)
Moderate (31–55 mm Hg)	12 (27.3)
Severe (> 55 mm Hg)	4 (90.9)

vMax = peak velocity.

Previously high gradient values (mean of all patients) in the aortic valve (mean: 46.88 mm Hg, max: 75.79 mm Hg) have been shown to decrease significantly after surgery (postoperative mean: 8.33 mm Hg, max: 16.10). The same is true for peak velocity (preoperative mean peak velocity (vMax): 4.25 m/sec, postoperative mean vMax: 1.90 m/sec) (Tables 4 and 5). According to these results, it is determined that there is a statistically significant decrease in mean, max gradients and vMax in the aortic valve (*P* < .001) (Table 6).

**Table 6:** Comparison of echocardiographic data.

	Preoperative	Postop 1st mo	*P* value
Aortic Mean Gradient (mm Hg), Median (Q1–Q3)	49.00 (41.00–55.00)	9.00 (5.00–11.00)	<.001*
Aortic Max Gradient (mm Hg), Mean ± SD	75.02 ± 25.90	16.10 ± 5.75	<.001**
Aortic vMax (m/sec), Median (Q1–Q3)	4.39 (4.00–4.60)	1.90 (1.69–2.20)	<.001*

vMax = peak velocity.

* Wilcoxon *t* test.

†Paired-samples *t* test.

## 4. Discussion

The literature recommends that stentless biological aortic valve prostheses are preferred to stented aortic valve prostheses for narrow aortic root valve replacement surgery. In this way, improvement in left ventricular functions and functional class is achieved.^[8]^ It is expected that the same effect will be valid in the Ozaki Procedure, as more valve area will be obtained compared to the valve prosthesis replacement. The Ozaki technique provides excellent results for effective orifis area and hemodynamically results comparable to that of natural, non-diseased aortic valve.^[9]^ When we compare the native aortic valve, Ozaki valve and prosthetic aortic valve, the native aortic valve and Ozaki valve are similar to each other in terms of hemodynamics. These 2 valves have a significantly larger orifice area than all prosthetic valves. When aortic valve flow increases, the native aortic valve and Ozaki valve show a similar increase in orifice area while prosthetic valves show a markedly weaker increase. In the same situation the native and Ozaki valve show a similar weaker increase in mean pressure gradient than prosthetic valves.^[10]^ The use of the AV-Neo method instead of traditional prosthetic valves, which significantly reduces the aortic valve area, jeopardizes the aortic root hemodynamically and causes foreign substance reaction, eliminates these disadvantages.^[11]^ Although the biological tissues degenerate in the Ozaki valve, the development of critical aortic stenosis may be delayed due to their effective orifis areas compared to prosthetic valves.^[9]^

The Ozaki Procedure can be applied in both aortic stenosis, aortic insufficiency and combined pathologies with tricuspid, bicuspid, and unicuspid aortic valves. Bicuspid and unicuspid valves can be transformed into a tricuspid form by forming a neo-commissure. The biggest disadvantage is that the annulus is properly measured and new valves implanted according to the created neo-commissures create proper coaptation. It is inevitable that the duration of cross-clamping will be longer.^[12]^ After the operation, serious decreases are provided in aortic valve gradient with values similar to native valve. A meta-analysis in 2023 for 1891 patients that underwent AV-Neo using the Ozaki technique provided that mean peak gradient was 15.7 mm Hg in aortic valve and had only %0.25 aortic valve insufficiency after operation.^[13]^ In our study, when the preoperative and postoperative first-month echocardiograms of the patients were compared, significant decreases were found in the mean, maximum gradients and vMax of the aortic valve. In addition, only 1 patient had severe aortic valve regurgitation that required postoperative reoperation. The results of a study, which included the short-term results of the Ozaki Procedure, which was first published in Europe and included 30 patients, were similar to the short-term results of literature and our study.^[14]^ In the study published by Ozaki in 2011, it was reported that the mean gradient in the aortic valve decreased by 19.0 ± 9.1 mm Hg at the end of the first week. In the same study, it was stated that the decrease in the gradient continued at the end of 1 year and its average was 12.9 ± 5.8 mm Hg.^[4]^ The early results of such studies in the literature support the early results of our study.

While valve repair technique is the gold standard in degenerative mitral regurgitation rather than mitral valve replacement,^[15]^ the Ozaki Procedure which is an aortic repair technique, is likely to become more popular than conventional replacement surgery for aortic valve surgery in the future. Besides, its biggest rival will be transcatheter aortic valve implantation (TAVI). However, the long-term results of TAVI are not encouraging. In a review of nearly 14,000 patients showed that, 5-year survival after TAVI is 48% in general population.^[16]^ Despite that the overall survival rate is 84.6% and freedom from reoperation is 95.8% 12 years after 1100 Ozaki Procedure.^[17]^ Autologous pericardium does not require prior material supply, it can be prepared immediately. Autologous pericardium is free and has no additional financial cost to the patient and hospitality cost. Autologous pericardium antigenicity is minimal and it does not induce a pronounced immune reaction.^[18]^ These are AV-Neo most important advantages over both TAVI and prosthetic valves.

As with any new technique for surgeons, the Ozaki Procedure technique has a learning curve.^[19]^ Although conventional sternotomy is usually required due to technical difficulties, there are also minimally invasive cases where upper sternotomy is performed.^[20]^ It may be considered discouraging by surgeons as it would take a longer time to learn compared to ring annuloplasty and traditional AVR. Even with more extensive surgeons, the cross-clamp time is longer in comparison with other aortic valve techniques. (Longer than 100–130 minutes).^[19]^ In our study, average cross-clamp time is 114 minutes in line with the literature.

## 5. Conclusion

AV-Neo may be a viable alternative to both surgical AVR and TAVI for AVR. Its popularity and application is increasing all over the world. Short and mid-term results are available in the literature. The short an mid-term results are good, and the long-term results are hopeful.

## Author contributions

**Conceptualization:** Yasin Ozden, Yavuz Sensoz.

**Data curation:** Yasin Ozden, Safa Ozcelik, Kemal Ozdemir, Ferdi Peynirci.

**Formal analysis:** Seyma Ozden, Yavuz Sensoz, Ilyas Kayacioglu.

**Investigation:** Yasin Ozden, Kemal Ozdemir, Ferdi Peynirci.

**Methodology:** Yasin Ozden, Seyma Ozden.

**Project administration:** Mutlu Senocak, Yavuz Sensoz, Ilyas Kayacioglu.

**Resources:** Safa Ozcelik, Ilyas Kayacioglu.

**Software:** Ferdi Peynirci, Mutlu Senocak, Ilyas Kayacioglu.

**Supervision:** Seyma Ozden, Mutlu Senocak, Yavuz Sensoz, Ilyas Kayacioglu.

**Validation:** Mutlu Senocak, Yavuz Sensoz.

**Visualization:** Safa Ozcelik, Seyma Ozden, Yavuz Sensoz.

**Writing – original draft:** Yasin Ozden.

**Writing – review & editing:** Yasin Ozden, Seyma Ozden.
